# Adjuvant endocrine therapy for postmenopausal breast cancer in the era of aromatase inhibitors: an update

**DOI:** 10.1186/1477-7800-3-31

**Published:** 2006-09-18

**Authors:** Ramia Mokbel, Isabella Karat, Kefah Mokbel

**Affiliations:** 1 St. George's Hospital, London, UK; 2 Professor at Brunel Institute of Cancer Genetics, Consultant Breast & Endocrine Surgeon. The Princess Grace Hospital, 42-52 Nottingham Place, London W1M 3FD, UK

## Abstract

There is overwhelming evidence that optimal adjuvant endocrine therapy for hormone sensitive breast cancer in postmenopausal women should include a third generation aromatase inhibitor (AI). On current evidence, adjuvant anstrozole or letrozole should be used upfront in such patients especially in those with high risk disease (node positive and/or tumours > 2 cm). The sequential approach of tamoxifen for 2–3 years followed by exemestane or anastrozole for 2–3 years is a reasonable alternative to 5 years of AI monotherapy in patients with low risk disease (node negative and tumour smaller than 2 cm) especially if the tumour is positive for estrogen and progesterone receptors.

Node-positive patients completing 5 years of adjuvant tamoxifen should be offered letrozole for up 48 months. Further research is required to establish the long-term cardiovascular safety of AIs especially that of letrozole and exmestane, the optimal AI to use, duration of AI therapy and whether monotherapy with an AI for 5 years is superior to sequencing an AI after 2–3 years of tamoxifen.

The bone mineral density (BMD) should be measured at baseline and monitored during therapy in women being treated with AIs. Anti-osteoporosis agents should such as bisphosphonates should be considered in patients at high risk of bone fractures.

## Background

Anti-aromatase drugs inhibit the cytochrome p-450 component of the aromatase enzyme complex responsible for the final step of estrogen biosynthesis in peripheral tissues. Third-generation aromatase inhibitors (anastrozole, letrozole, and exemestane) are now considered the gold standard endocrine therapy in the first-line and second-line settings for estrogen receptor (ER) and/or progesterone receptor (PgR) positive advanced breast cancer in postmenopausal women [1]. Furthermore there is a growing body of evidence for their superiority to tamoxifen in the adjuvant setting. The latter is the focus of this article.

Several studies have shown that aromatase inhibitors (AIs) are superior to tamoxifen in the adjuvant setting for postmenopausal women with ER positive breast cancer during the first 5 years.

## Anastrozole

The ATAC (Arimidex, Tamoxifen, Alone or in Combination) study [2] has shown that 5 years of anastrozole is superior to tamoxifen in terms of efficacy and tolerability in treating postmenopausal women with ER positive breast cancer. After a median follow-up of 68 months, (9366 postmenopausal women with localised breast cancer), anastrozole significantly prolonged disease-free survival (DFS) and reduced the risk of recurrence by 26% in patients with hormone-receptor positive disease [hazard ratio = 0.74, 95% CI 0.64–0.87, p = 0.0002]. There was a 12% statistically non-significant reduction in breast cancer-related deaths in the anastrozole group compared to tamoxifen and a reduction of borderline significance in distant metastases (hazard ratio: 0.84, p = 0.056). However, no significant difference in overall survival (OS) was seen after 68 months. This may be due to the fact that the follow-up interval is currently too short to see such a difference. However, another potential contributing factor to the similarity in OS is the increase in non-breast cancer deaths in the anastrozole group. The latter was largely due to an excess of new non-breast cancers (statistically non-significant). There was no significant difference between the two groups in relation to the incidence of cardiac mortality, myocardial infarction (MI) or ischaemic heart disease (IHD) suggesting no adverse cardiac effect for anastrozole compared with tamoxifen which is considered cardio-protective. The incidence of deep venous thrombosis (DVT) [1.6% vs. 2.4%, p = 0.02], stroke (odds ratio = 0.7; 95% CI = 0.50–0.97), contralateral breast cancer and endometrial tumours was lower in women taking anastrozole compared with tamoxifen. However, anastrozole use was associated with a higher incidence of hypercholesterolemia, arthralgia, osteoporosis and fractures (11.0% vs. 7.7%, p < 0.0001) compared with tamoxifen. The latter observation underscores the importance of determining BMD at baseline and monitoring it during therapy. Anti-osteoporosis agents such as bisphosphonates can be used for prevention or treatment as required.

The ATAC study [2] has therefore recommended a 5-year initial course of anastrozole instead of tamoxifen especially in women with ER+ve and PgR-ve breast cancer. This recommendation has been also based on the observation that the hazard of recurrence is highest in the first two years especially in patients with node positive disease and the benefit is greatest in women with ER+ve and PgR-ve breast cancer [3]. Surprisingly however no significant benefit was seen in node positive patients and those who had received chemotherapy in subgroup analyses. Furthermore, the observation that women with ER+ve and PgR-ve breast cancer derive a greater benefit from adjuvant AI therapy has not been replicated in other adjuvant AI trials.

## Letrozole

The Breast International Group (BIG) 1–98 study is a randomized, phase III, double-blind trial comparing five years of treatment with various adjuvant endocrine therapy regimens in postmenopausal women with hormone-receptor-positive breast cancer: letrozole, letrozole followed by tamoxifen, tamoxifen, and tamoxifen followed by letrozole [4]. The analysis comparing the two groups assigned to receive letrozole initially with the two groups assigned to receive tamoxifen initially has been recently published. A total of 8010 women with assessable data were enrolled, 4003 in the letrozole group and 4007 in the tamoxifen group. After a median follow-up of 25.8 months, the five-year DFS estimates were 84.0% and 81.4%, respectively. Letrozole significantly reduced the risk of recurrence by 28% with hormone-receptor positive disease (hazard ratio = 0.72; 95% CI = 0.61 to 0.86; P < 0.001), especially the risk of distant recurrence (hazard ratio = 0.73; 95% CI, 0.60 to 0.88; P = 0.001). This risk reduction of distant relapse is greater than that seen with anastrozole in the ATAC study, however caution should be exercised when considering such a cross-trial comparison. Thromboembolism, endometrial cancer, contralateral breast cancer and vaginal bleeding were more common in the tamoxifen group. Women given letrozole had a higher incidence of fractures (5.7% vs. 4.0%, p < 0.001) indicating the need for the measurement and monitoring of BMD. Furthermore the incidence of cardiovascular events (excluding thrombo-embolism) such as grade 3–5 cardiac failure (2.1% vs. 1.1%, p = 0.0003) and hypercholesterolemia was also higher among women given letrozole. Therefore longer follow up is required in order to assess the long term safety of letrozole especially regarding the cardiovascular system. The incidence of cerebro-vascular events (1%) was similar in both groups.

Unlike the ATAC study, subgroup analyses of BIG1-98 showed a significant DFS benefit in favour of letrozole among high risk groups such as patients with node positive breast cancer and/or tumours larger than 2 cm. Furthermore, the letrozole benefit was seen in all ER+ cases regardless of PgR status highlighting the limitations of subgroup analyses in large trials.

## The switching/sequencing approach

Two recent studies [5,6] which switched patients from tamoxifen to an AI after 2–3 years showed a significant improvement in 5-year DFS. In the well-designed International Exemestane Study (IES) which recruited 4742 patients, the adjusted hazard ratio for DFS was 0.63 (95% CI = 0.51–0.77, p = 0.00001) in favour of exemestane, suggesting that patients benefit from using both drugs sequentially. The distant DFS but not OS was also significantly better in the exemestane arm compared with tamoxifen (HR = 0.66, p = 0.00004, HR = 0.88, p = 0.37 respectively). Osteoporosis, visual disturbances and arthralgia were more frequently seen with exemestane (p = 0.05, 0.04 and 0.01 respectively) whereas thromboembolism, vaginal bleeding, contralateral breast cancer and other non-breast primary cancers were more frequent in the tamoxifen arm. Although the incidence of MI was increased in the exemestane group compared with tamoxifen arm (20 vs. 8 MIs, p = 0.023), there was difference in cardiac mortality between the two groups. An update of the IES has been recently presented at The American Society of Clinical Oncology (June 2006). After a median a follow up of 4.8 years, there was no statistically significant OS benefit in favour of the switch as initially predicted (OS benefit = 15%, p = 0.08).

Jonat et al [7] have recently analyzed patient data on 4,009 postmenopausal women enrolled in three studies – Arimidex-Nolvadex (tamoxifen) 95 (ARNO 95), the Austrian Breast & Colorectal Cancer Study Group 8 (ABCSG-8), and the Intergroup Tamoxifen Anastrozole (ITA) trial. All of the studies showed benefits in terms of DFS for women who had switched to Arimidex after two or three years of tamoxifen. The meta-analysis (the median follow-up on all the studies was 30 months), though, showed an OS advantage in favour of the switch. The hazard ratio for death was 0.71 with a 95% confidence interval ranging from 0.52 to 0.98 for the meta-analysis data. The meta-analysis also showed that the group that switched had a 41% improvement in DFS (hazard ratio = 0.59, p-value less than 0.0001) and significantly longer time to any recurrence.

It is important to note that both switch studies' patients were disease-free at the time of randomisation to either continued tamoxifen or an AI (i.e. disease-free at 2–3 years of follow-up), thus making any direct comparison with AI monotherapy trials invalid.

The potential advantages of the switching approach include the bone protection and cholesterol lowering effect conferred by tamoxifen prior to starting an AI. It remains to be proven whether a sequencing approach will lead to a reduction in fractures and ischaemic heart disease. Data from the bone sub-protocol of the IES show that the gain in bone mineral density (BMD) in patients treated with tamoxifen for 2–3 years is lost rapidly after starting an AI. Differences in BMD appear within six months of switching, and the loss is similar to that seen with other AIs at 2–3% in the first year of therapy with exemestane. Such observations underscore the importance of BMD monitoring and preventative intervention in patients taking an AI.

Using Markov models to simulate 10-year DFS among postmenopausal women with ER positive breast cancer, Punglia et al [8], analysed three treatment strategies: 5 years of tamoxifen alone, 5 years of an AI alone, and sequential treatment consisting of tamoxifen with cross over to an AI at 2.5 or 5 years. The authors found that sequential therapy with tamoxifen followed by cross over to an AI at 2.5 years yielded a significant improvement in DFS compared with planned AI monotherapy. At 10 years, the cross-over strategy achieved absolute DFS rates of 83.7% and 67.6% for node-negative and node-positive patients, respectively, compared with 82.6% and 65.5%, respectively, for AI monotherapy, which is a 6% relative risk reduction. The DFS improvement was apparent after 6 years. Later cross over from tamoxifen to an AI at 5 years did not further improve 10-year DFS estimates. This analysis suggests that sequential treatment could be superior to AI mono-therapy in terms of DFS. However, this model used heterogeneous end-points from different trials and assumed constant recurrence rates.

Contradicting conclusions were reached by a different model constructed by Cuzick et al [9] who concluded that the switching strategy will be always inferior to 5 years of AI monotherapy. This model assumed phenotypic receptor remodelling and a constant benefit from AIs over 10 years.

Since such models are based on certain assumptions, the question whether 5 years of AI monotherapy is superior or inferior to sequential teerapy approach can be only answered by RCTs. Results from the ongoing arms of the Big 1–98 study, which are expected to determine whether mono-therapy or sequential therapy is more effective, and if sequential therapy, which sequence is more effective, are expected in 2008.

## Extended adjuvant

Furthermore postmenopausal women completing 5 years of adjuvant tamoxifen, letrozole has been shown to be of value in reducing breast cancer recurrence (p < 0.0001) when given in the extended adjuvant therapy (up to 48 months of treatment) setting in the MA.17 study [10]. The latter is a double-blind placebo-controlled trial involving 5187 postmenopausal women who had completed 5 years of tamoxifen. Furthermore, this study showed a significant OS benefit in patients with node-positive disease (n = 2360, p = 0.038).

## Contralateral breast cancer

It should be noted that all three AIs were found to be superior to tamoxifen in reducing the risk of contralateral breast cancer [2,4,5] indicating that tamoxifen is a suboptimal chemopreventative strategy.

## Costeffectiveness

Finally in relation to cost-effectiveness, it has been estimated that tamoxifen for 2–3 years followed by an AI achieves the lowest cost/QALY estimates, a further improvement of DFS of 1% if the AI is given up front provides an acceptable cost/QALY. However, the additional benefits achieved by administering an AI subsequent to 5 years of tamoxifen seem to provide unacceptable costs [11].

## Conclusion

• Optimal adjuvant endocrine therapy for hormone sensitive breast cancer in postmenopausal women should include an AI.

• Adjuvant anstrozole or letrozole should be used upfront in such patients especially in those with high risk disease (node positive and/or tumours > 2 cm).

• The sequential approach of tamoxifen for 2–3 years followed by exemestane or anastrozole for 2–3 years is a reasonable alternative to 5 years of AI monotherapy in patients with low risk disease (node negative and tumour smaller than 2 cm) especially if the tumour is positive for estrogen and progesterone receptors.

• Node-positive patients completing 5 years of adjuvant tamoxifen should be offered letrozole for up 48 months.

• The bone mineral density (BMD) should be measured at baseline and monitored during therapy in women being treated with AIs. Anti-osteoporosis agents should such as bisphosphonates should be considered in patients at high risk of bone fractures.

• Further research is required to establish the long-term cardiovascular safety of AIs especially that of letrozole and exmestane, the optimal AI to use, duration of AI therapy and whether monotherapy with an AI for 5 years is superior to sequencing an AI after 2–3 years of tamoxifen.
